# Regulation of mitochondrial cargo-selective autophagy by posttranslational modifications

**DOI:** 10.1016/j.jbc.2021.101339

**Published:** 2021-10-22

**Authors:** Anna Lechado Terradas, Katharina I. Zittlau, Boris Macek, Milana Fraiberg, Zvulun Elazar, Philipp J. Kahle

**Affiliations:** 1 Laboratory of Functional Neurogenetics, Department of Neurodegeneration, Hertie Institute for Clinical Brain Research, University of Tübingen, Tübingen, Germany; 2 Interfaculty Institute of Biochemistry, University of Tübingen, Tübingen, Germany; 3 Proteome Center Tübingen, University of Tübingen, Tübingen, Germany; 4 Department of Biomolecular Sciences, The Weizmann Institute of Science, Rehovot, Israel; 5 German Center for Neurodegenerative Diseases (DZNE), Tübingen, Germany

**Keywords:** autophagy, mitochondria, protein kinase PINK1, phosphorylation, ubiquitin ligase parkin, ubiquitylation, AMBRA1, activating molecule in beclin-1 regulated autophagy protein 1, AMPK, AMP-activated protein kinase, ARIH1, ariadne-1 homolog in humans, ATG, autophagy-related protein, BAD, Bcl-2 associated agonist of cell death, BAK, Bcl-2 homologous antagonist/killer, Bcl, B cell lymphoma, BH3, Bcl-2 homology 3 domain, BNIP, Bcl-2 19 kDa protein-interacting protein, CCCP, carbonyl cyanide *m*-chlorophenyl hydrazone, CLEAR, coordinated lysosomal expression and regulation, DFCP1, double FYVE domain-containing protein 1, ΔΨ_m_, mitochondrial membrane potential, DRP1, dynamin-related protein 1, DUB, deubiquitylating enzyme, Far, factor arrest, FIP200, focal adhesion kinase-interacting protein of 200 kDa, FIS1, mitochondrial fission 1 protein, FKBP8, FK506-binding protein 8, FUNDC1, FUN14 domain-containing protein 1, GABARAP, γ-amino-butyric acid receptor-associated protein, GCN5L1, general control of amino acid synthesis protein 5-like 1, gp78, glycoprotein 78, HK2, hexokinase 2, HOPS, homotypic fusion and protein sorting, HUWE1, homologous to the E6-AP carboxyl terminus (HECT), ubiquitin-associated and WWE domain-containing protein 1, LC3, microtubule-associated protein light chain 3, LIR, LC3-interacting region, Mcl-1, induced myeloid leukemia cell differentiation protein, MFF, mitochondrial fission factor, MFN, mitofusin, MGRN1, mahogunin RING finger protein 1, MIM, mitochondrial inner membrane, MIRO, mitochondrial Rho GTPase, MITOL, mitochondrial ubiquitin ligase, MOM, mitochondrial outer membrane, mTOR, mammalian target of rapamycin, mTORC1, mTOR complex 1, MTS, mitochondrial targeting sequence, MUL1, mitochondrial ubiquitin ligase 1, NBR1, next to breast cancer type 1 susceptibility gene 1 protein, NDP52, nuclear dot protein of 52 kDa, NF-κB, nuclear factor-κB, NGLY1, N-glycanase 1, OMA1, overlapping with the mitochondrial matrix ATPase associated with diverse cellular activities (m-AAA) protease 1, OPA1, optic atrophy protein 1, PARL, presenilin-associated rhomboid-like protease, PD, Parkinson's disease, PI3P, phosphatidylinositol 3-phosphate, PINK1, phosphatase and tensin homolog (PTEN)-induced kinase 1, PTM, post-translational modification, RAB, Ras-associated binding protein, RING, really interesting new gene, SIRT, sirtuin, SNARE, soluble N-ethylmaleimide-sensitive factor-attachment protein receptor, SUMO, small ubiquitin-related modifier, TBK1, tumor necrosis factor receptor-associated factor family member associated NF-κB activator-binding kinase 1, TFEB, transcription factor EB, TOM, translocase of the outer membrane, Ubl, ubiquitin-like domain, ULK1, uncoordinated protein 51-like kinase 1, USP, ubiquitin-specific protease, VDAC, voltage-dependent anion selective channel, VPS, vacuolar sorting protein, WIPI, WD40 repeat protein interacting with phosphoinositides

## Abstract

Mitochondria are important organelles in eukaryotes. Turnover and quality control of mitochondria are regulated at the transcriptional and posttranslational level by several cellular mechanisms. Removal of defective mitochondrial proteins is mediated by mitochondria resident proteases or by proteasomal degradation of individual proteins. Clearance of bulk mitochondria occurs *via* a selective form of autophagy termed mitophagy. In yeast and some developing metazoan cells (*e.g.*, oocytes and reticulocytes), mitochondria are largely removed by ubiquitin-independent mechanisms. In such cases, the regulation of mitophagy is mediated *via* phosphorylation of mitochondria-anchored autophagy receptors. On the other hand, ubiquitin-dependent recruitment of cytosolic autophagy receptors occurs in situations of cellular stress or disease, where dysfunctional mitochondria would cause oxidative damage. In mammalian cells, a well-studied ubiquitin-dependent mitophagy pathway induced by mitochondrial depolarization is regulated by the mitochondrial protein kinase PINK1, which upon activation recruits the ubiquitin ligase parkin. Here, we review mechanisms of mitophagy with an emphasis on posttranslational modifications that regulate various mitophagy pathways. We describe the autophagy components involved with particular emphasis on posttranslational modifications. We detail the phosphorylations mediated by PINK1 and parkin-mediated ubiquitylations of mitochondrial proteins that can be modulated by deubiquitylating enzymes. We also discuss the role of accessory factors regulating mitochondrial fission/fusion and the interplay with pro- and antiapoptotic Bcl-2 family members. Comprehensive knowledge of the processes of mitophagy is essential for the understanding of vital mitochondrial turnover in health and disease.

## Mitochondria, development, aging, and Parkinson's disease (PD)

Mitochondria are specialized organelles present in most eukaryotic cells. In addition to energy production, mitochondria play important roles in nutrient and lipid metabolism as well as in apoptosis. Two specialized membranes compartmentalize mitochondria. This feature, together with remnants of an own genome (mitochondrial DNA; mtDNA), is likely due to their endosymbiont heritage. Although the vast majority of 1136 proteins in human mitochondria (www.broadinstitute.org/files/shared/metabolism/mitocarta/human.mitocarta3.0) are encoded in the nucleus, the mitochondrial genome codes for 13 proteins, all of which are components of the respiratory chain (1). During oxidative phosphorylation, electrons flow through the respiratory chain in the mitochondrial inner membrane (MIM). This process builds up the membrane potential (proton gradient) that drives ATP synthase. As a by-product, reactive oxygen species are produced (2). Oxidative stress and mtDNA damage are associated with age-related mitochondrial dysfunction in a complex manner (3). As their energy production is obligatorily dependent on oxidative phosphorylation, neurons are particularly vulnerable to mitochondrial dysfunctions. Oxidative damage is aggravated in age-dependent neurodegenerative diseases, such as PD (4, 5). Indeed, the two most common recessive PD gene products, phosphatase and tensin homolog (PTEN)-induced kinase 1 (PINK1) and the E3 ubiquitin ligase parkin, are enzymes that mediate the autophagic removal of mitochondria (mitophagy). This mitophagy pathway might offer therapeutic targets for the treatment of PD (6). Moreover, mitochondrial turnover is important for cellular homeostasis, and in a few cell types (reticulocytes, germ cells) mitochondria are eliminated during normal development.

Given the tremendous importance of mitophagy in development, aging, and neurodegeneration, it is imperative to understand the cellular mechanisms regulating cargo-selective autophagy of mitochondria. Enormous progress in understanding this process in diverse cells types has been made in the past two decades, unraveling key players including the aforementioned PINK1, parkin, and other mitochondrial outer membrane (MOM) ubiquitin ligases as well as ubiquitin-binding autophagy adaptors. Clearly, mitophagy is embedded in an intricate signaling network integrating mitochondrial elimination signals, complex posttranslational modifications (PTMs) of MOM proteins, and the coupling to autophagy propagation toward autophagolysosomal degradation. These complex signaling events throughout the entire process of mitophagy are beginning to be understood, also thanks to recent proteomic studies. This review describes how the basic elements of the autophagy machinery and their coupling to cargo-selective mitochondrial removal are connected *via* PTMs mediated by mitophagy-regulating enzymes.

## Brief introduction to autophagy

Macroautophagy (hereafter autophagy) is a major intracellular, tightly regulated catabolic process in eukaryotes that delivers cytosolic components including protein aggregates, damaged organelles, and invasive pathogens for lysosomal degradation (7). This process is activated in response to various stressful conditions such as nutrient starvation, growth factors deprivation, hypoxia and infections, and is essential for maintenance of cellular homeostasis (7, 8). For example, autophagy supplies nutrients and energy for vital anabolic cellular functions during fasting and other stresses (9). The core mechanism comprises the sequestration of “to-be-degraded” cargo into the cup-shaped isolation membrane termed phagophore, which expanses and seals into a double-membraned sphere-termed the autophagosome- which engulfs autophagic cargo. Upon maturation, the outer membrane of autophagosome fuses with the vacuole (in yeast and plants) or endosomes and lysosome (in metazoans), leading to the degradation of the autophagic body together with its cargo by lysosomal catabolic enzymes (7, 8, 10).

**Figure 1:**
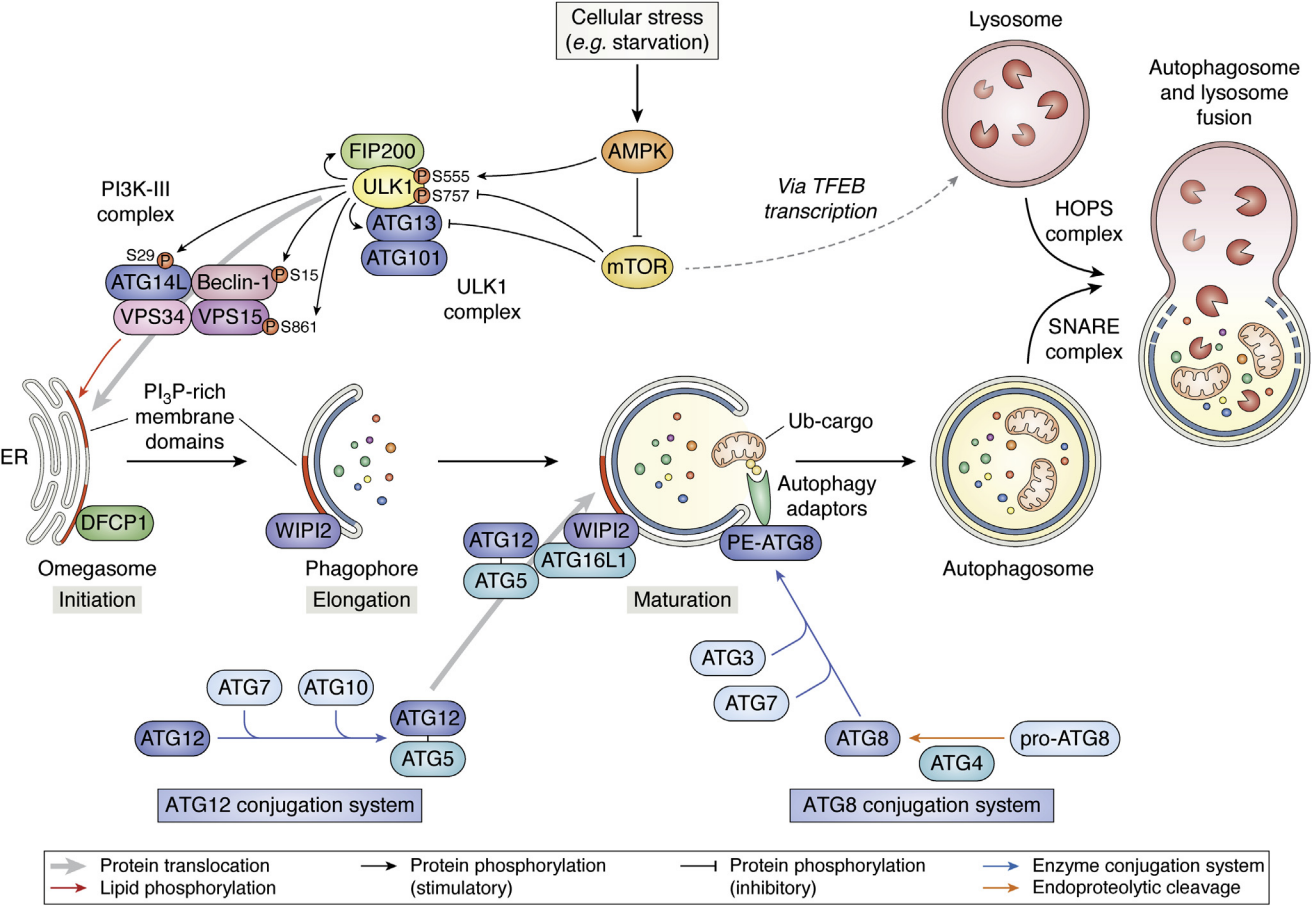
**Key elements of the autophagy pathway.** Cellular stress (*i.e.*, starvation) is sensed by AMPK, which directly activates the ULK1 complex by a complex phosphorylation pattern, including S555, and relieves it from the mTOR block by suppressing ULK1 S757 phosphorylation. The ULK1 kinase phosphorylates several components of the ULK1 megacomplex as well as the PI3K-III complex, promoting its translocation to the ER, where it generates membrane domains rich in PI3P that are eventually recognized by PI3P-binding proteins which promote phagophore formation. Two enzyme conjugation systems mediate phagophore maturation and insert lipidated ATG8 (or LC3-II) as anchors for autophagy receptors/adaptors that couple to ubiquitylated cargo. Finally, autophagosomes fuse with lysosomes (or the vacuole in yeast) *via* interaction of the SNARE and HOPS complexes, respectively. See text for details. *Thick gray arrows* indicate protein translocations, *thin black arrows* indicate protein phosphorylations (*arrowheads* stimulatory, *hammerheads* inhibitory), and the *thin red arrow* indicates lipid phosphorylation. *Blue arrows* indicate enzyme conjugation systems, and the *orange arrow* endoproteolytic cleavage. The *dashed line* indicates an indirect transcriptional (CLEAR) pathway for lysosome biogenesis.

Autophagy is activated by cellular triggers such as amino acid deprivation, which inhibits the master cell growth regulator serine/threonine kinase “mammalian target of rapamycin” (mTOR), and reduced energy levels activating AMP-activated protein kinase (AMPK) (11). In high nutrient conditions, the mTOR complex 1 (mTORC1) binds and phosphorylates the uncoordinated protein 51-like kinase 1 (ULK1) at residue S757, which disrupts the interaction with inactive AMPK (12). Upon starvation, AMPK is activated, which blocks mTORC1 activity and initiates autophagy at the transcriptional level through dephosphorylation of the master transcription factor EB (TFEB) that is negatively regulated by mTORC1 (Fig. 1). Dephosphorylated TFEB is released from mTORC1 and translocates to the nucleus where it stimulates coordinated lysosomal expression and regulation (CLEAR) by binding to the CLEAR-box sequence (5′-GTCACGTGAC-3′) present in the regulatory region of many lysosomal and autophagy-associated genes (13). Moreover, inactive mTORC1 dissociates from the ULK1 complex, which reduces ULK1 S757 phosphorylation. Instead, starvation-activated AMPK binds to ULK1 and phosphorylates it on multiple distinct sites (12). The complex phosphorylation pattern of AMPK-activated ULK1 is not fully understood, but S555 is a prominently phosphorylated residue upon starvation and mitophagy induction (14).

Activated ULK1-bearing numerous PTMs forms an oligomeric megacomplex together with its cofactors, autophagy-related protein ATG13, focal adhesion kinase-interacting protein of 200 kDa (FIP200) and ATG101, which is translocated to ER-associated isolation membrane assembly sites (15, 16). The active ULK1 megacomplex is targeted to the isolation membrane *via* ATG14L (15). ATG14L together with beclin-1 associates with the lipid kinase vacuolar protein sorting-associated protein VPS34 and the pseudokinase VPS15 to form a PI3K-III (Fig. 1). The ULK1 complex component ATG13 targets ULK1 to its substrate ATG14L for S29 phosphorylation (17) and the ATG14L partner beclin-1 for S15 phosphorylation (18). In addition, a recent phosphoproteomic study identified VPS15 S861 as a major regulatory ULK1 target site that activates the VPS34 PI3K activity (19). Such regulation of PI3K promotes the local production of phosphatidylinositol 3-phosphate (PI3P) at an ER structure called “omegasome” (Fig. 1), recruiting the PI3P-binding double FYVE domain-containing protein DFCP1 (20). Subsequent binding of the WD40 repeat protein interacting with phosphoinositides WIPI2 (a mammalian otholog of yeast Atg18) mediates omegasome maturation into phagophores (21).

Phagophore elongation requires two conjugation systems, involving the E1 ubiquitin ligase ATG7 and the E2 ubiquitin-conjugating enzyme ATG10, which links the ubiquitin-like protein ATG12 to ATG5 (Fig. 1). ATG16L1 is recruited to the phagophore *via* binding to WIPI2 and conjugated to ATG5-ATG12, forming an ATG5-ATG12-ATG16L1 complex (7, 22). The other conjugation system targets the ATG8 proteins, which can be subgrouped into LC3 (microtubule-associated protein light chain 3) and GABARAP (γ-amino-butyric acid receptor-associated protein) family members. Nascent pro-ATG8 precursors are processed by ATG4 cysteine proteases. The proteolytically processed ATG8 proteins expose a C-terminal glycine, allowing conjugation to phosphatidylethanolamine by the ATG3 E2 conjugating enzyme after activation by ATG7 (Fig. 1). Such ATG8 lipidations promote phagophore expansion (7, 15). Lipidated ATG8 proteins play an important role in the recruitment of cargo destined to lysosomal degradation into autophagosome, a process mediated by autophagy receptors, which brings part of the machinery to the autophagosomal membrane. The ULK1 complex regulates the membrane supply for sealing and involves the ATG9 trafficking system. Additionally, ULK1 may also phosphorylate various autophagy receptors, thus playing a role in selective autophagy including mitophagy (23, 24, 25) (for more details, see below).

Following phagophore sealing, the autophagosome undergoes maturation that results in clearance of ATGs from the autophagosomal outer membrane and recruitment of two machineries: one responsible for lysosomal delivery, comprised of kinesin motor; and the other responsible for autophagosome fusion with the lysosome, comprised of a soluble N-ethylmaleimide-sensitive factor-attachment protein receptor (SNARE), including syntaxin17 and synaptosomal-associated protein 29 on the autophagosome and vesicle-associated membrane protein 8 on the lysosome (Fig. 1). The lysosomes are recruited and tethered to autophagosomes *via* the homotypic fusion and protein-sorting (HOPS) complex, which is composed of VPS11, VPS16, VPS18, VPS33A, VPS39, and VPS41 (26, 27). The HOPS complex interacts with the SNAREs and facilitates fusion of autophagosome with the lysosome (28).

Whereas bulk autophagy mediates a nonspecific engulfment and degradation of cytoplasmic material, selective autophagy is mediated by specific autophagy receptors. The main role of selective autophagy is to maintain the intracellular homeostasis by selectively degrading distinct cellular components such as damaged mitochondria (mitophagy), endoplasmic reticulum (ERphagy), lysosomes (lysophagy) invading pathogens (xenophagy), aggregated proteins (aggrephagy), and others (7, 29). The molecular machinery of selective autophagy must efficiently identify the cargo and sequester it within autophagosomes. Autophagic receptors/adaptors that contain ubiquitin-interacting motifs (*e.g.*, p62 sequestosome-1, optineurin, nuclear dot protein of 52 kDa (NDP52), next to breast cancer type 1 susceptibility gene 1 protein (NBR1) and autophagy-linked FYVE protein) connect their ubiquitylated cargo with the autophagosome *via* LC3-interacting region (LIR) motifs (29, 30). On the other hand, autophagy receptors such as the B cell lymphoma-2 (Bcl-2) 19 kDa protein-interacting proteins BNIP3 and BNIP3L/NIX or the FUN14 domain-containing protein 1 (FUNDC1) act independently of ubiquitin to deliver cargo to phagophores *via* their LIR motifs directly (30, 31).

## General principles of cargo-selective mitochondrial autophagy

Mitophagy is the cellular catabolic process responsible for maintenance of mitochondrial homeostasis and quality control. Various developmental, environmental, physiological, and pathological stimuli activate mitophagosome formation resulting in mitochondrial clearance (32). Here we discuss cargo-associated PTMs that activate mitophagy.

### Regulatory mechanisms of mitophagy in yeast

Activation of mitophagy in yeast may be achieved by their growing in nonfermentable medium or by nitrogen starvation (33). Atg32 was identified as an outer mitochondrial membrane receptor essential for mitophagy (34). Atg32 directly interacts with Atg8 and the adaptor protein Atg11 (35). While the Atg11-Atg32 interaction is crucial for mitochondria recognition as a cargo and recruits the targeted mitochondria to the phagophore assembly site, the Atg8-Atg32 interaction through Atg32 LIR motif mediates phagophore expansion. The transcriptional levels of Atg32 may be upregulated due to oxidative stress or starvation conditions *via* suppression of TOR and release of transcriptional suppression of Atg32 by the Ume6-Sin3-Rpd3 complex (36). However, the main regulation of Atg32 is by posttranscriptional phosphorylation at S114 and S119 (37). Such phosphorylation facilitates Atg32 interaction with Atg11. Especially, phosphorylation at S114 is essential for successful mitophagy, since substitution of this serine residue to alanine prevents Atg11-Atg32 interaction and abolishes mitophagy (37). Regulation of mitophagy by phosphorylation and dephosphorylation of Atg32 is mediated by balanced activity of casein kinase 2 and protein phosphatase 2A complexed with factor arrest proteins (38).

### Regulatory mechanisms of mitophagy in mammalian cells

Two pathways are responsible for mitophagy mediation in mammalian cells, the ubiquitin-mediated pathway and receptor-mediated pathway. The ubiquitin-mediated pathway is dependent on autophagy adaptors/receptors such as p62, optineurin, transient axonal glycoprotein 1 binding protein 1, NDP52, and NBR1 that contain ubiquitin-binding domains in addition to their LIR motif (30). These adaptors/receptors sequester the ubiquitylated mitochondrial proteins through their ubiquitin-binding domain and recruit the mitochondria into autophagosomes by interacting with lipidated ATG8s through their LIR motifs (39). The ubiquitylation pathway is mainly mediated by the E3 ubiquitin ligase, parkin (40). Upon mitochondrial depolarization, PINK1 accumulates on the outer membrane of damaged mitochondria and recruits parkin (41), which ubiquitylates MOM proteins and induces mitophagy. On the other hand, parkin ubiquitylation-independent mitophagy pathways are mediated by autophagy receptors such as NIX, BNIP3, FUNDC1, Bcl-2-like protein 13, and FK506-binding protein 8 (FKBP8) (30, 42). All of these receptors undergo MOM incorporation and recruit lipidated ATG8s *via* their LIR motif. Such recruitments label damaged mitochondria as a cargo and promote their engulfment into the growing phagophore.

In addition to protein receptors that mediate mitophagy, several lipids are also implicated in this process. Cardiolipin was reported to act as mitophagy receptor in neuronal cells. Upon mitochondrial stress, cardiolipin translocates from the MIM to the MOM and there, directly interacts with the N-terminus of LC3, mediating clearance of damaged mitochondria (43). Ceramide may also directly interact with lipidated LC3B on mitochondrial membrane, mediating mitophagy (44).

Removal of mitochondria *via* autophagy generally occurs under two conditions (45): (i) quality control removing dysfunctional mitochondria to maintain cellular homeostasis and viability (see below) and (ii) during organismal development, for example, when red blood cells lose all their organelles in the terminal step of erythropoiesis. Upregulation of the autophagy receptor NIX during terminal erythroid differentiation is the key trigger for cargo-selective autophagic removal of mitochondria from red blood cells (46, 47). A portion of mitochondria may lose membrane potential (ΔΨ_m_) (46), which could stimulate stress-induced selective mitochondrial recruitment of membrane-conjugated ATG8 proteins *via* NIX (48). However, alternative macroautophagy independent of the ATG8 lipidation factors ATG5 and ATG7 is suggested as the major pathway for mitochondrial clearance during development (49). This alternative pathway involves the previously reported mitophagy effector ULK1 (50). The detailed mechanisms that govern mitochondrial autophagy in erythrocyte differentiation remain to be elucidated.

## PINK1/parkin-dependent mitophagy

**Figure 2:**
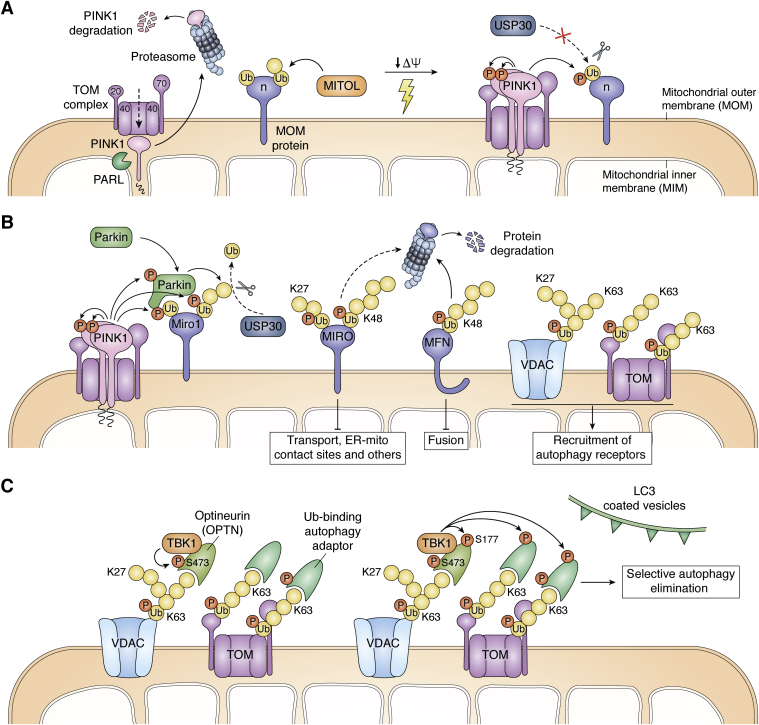
**Schematic representation of parkin-dependent mitophagy time course.***A*, in normal conditions, PINK1 is imported and partially cleaved by the mitochondrial protease PARL, after which PINK1 gets eliminated by the UPS following the N-end rule, leading to very low endogenous PINK1 levels in healthy conditions. MITOL prepares a ubiquitin coat on MOM substrates. Upon mitochondrial membrane depolarization, the protein import machinery gets compromised and PINK1 is accumulated at the MOM. PINK1 gets auto-phosphorylated, and this triggers its activation: PINK1 starts phosphorylating ubiquitin molecules that were previously attached to MOM substrates, antogonizing USP30-mediated ubiquitin removal of those chains. *B*, phosphorylation of ubiquitin by PINK1 triggers parkin recruitment to the MOM. Direct PINK1 phosphorylation of the parkin Ubl and the binding to phosphorylated ubiquitin trigger a conformation change of parkin, which activates its E3-ubiquitin ligase potential. As a result, diversely linked ubiquitin chains are built on MOM substrates. Proteins involved in mitochondrial transport and fusion are mostly polyubiquitylated with K48-linked chains and are rapidly eliminated *via* the ubiquitin-proteasome system. MIRO1/2 is also ubiquitylated through K27-linked chains and is involved in multiple functions other than mitochondrial transport. Differential linkages might explain the remaining forms of ubiquitylated MIRO1/2 after mitochondria depolarization (*dashed arrow*). Other MOM proteins such as VDAC2 and TOM20 are mostly ubiquitylated with K63-linked and K27-linked chains, which triggers the recruitment of autophagy receptors. Ubiquitin chain building may be counteracted by USP30. *C*, S473 within the UBL domain of optineurin is phosphorylated by bound TBK1 when recruited to ubiquitylated MOM proteins, further enhancing optineurin recognition of ubiquitin chains. TBK1 also phosphorylates optineurin and additional autophagy receptors within the LIR motif (S177 in optineurin), which increases the affinity for lipidated ATG8/LC3 molecules and eventually triggers the recruitment of mitochondria to autophagosomes for their final elimination.

The discovery of mitophagy controlled by the two most common recessive PD genes encoding PINK1 and parkin (40, 51, 52, 53) offered tremendous insights into the cell biology of mitochondrial quality control. Under normal conditions, PINK1 is effectively transported into mitochondria, where it is immediately degraded. As the mitochondrial protein import machinery depends on ΔΨ_m_, damaged mitochondria with collapsed ΔΨ_m_ can no longer import PINK1. Instead, PINK1 massively accumulates on the MOM (41, 54). In this localization, PINK1 faces cytosolic kinase substrates, most notably ubiquitin and the parkin ubiquitin-like domain (Ubl) (55, 56, 57). The PINK1-catalyzed S65 phosphorylation of ubiquitin and parkin Ubl activate parkin E3 ubiquitin ligase activity (58, 59) toward mitochondrial substrates, targeting depolarized mitochondria for autophagic degradation (Fig. 2).

### PINK1-dependent phosphorylations

Under healthy conditions, PINK1 is imported into mitochondria *via* an N-terminal mitochondrial targeting sequence (MTS). Once imported into the mitochondrial matrix, the MTS of pre-PINK1 is cleaved off by the mitochondrial processing protease (60). PINK1 then accesses the inner membrane active site of presenilin-associated rhomboid-like protease (PARL), which cleaves PINK1 within the membrane-anchoring segment (61, 62). In healthy cells, processed PINK1 is retrotranslocated into the cytosol for proteasomal degradation within 1 h (63, 64) (Fig. 2*A*). In most cell types, steady-state levels of PINK1 are practically undetectable in the absence of stress, but this does not rule out potential physiological functions of PINK1 (65, 66), which remain poorly understood to date.

When the kinase PINK1 cannot be imported for mitochondrial processing and clearance, it accumulates on depolarized mitochondria and thus acts as a molecular switch to trigger mitophagy. Following such accumulation at the MOM, PINK1 becomes autophosphorylated at S228 and S402, which is important for PINK1 catalytic activity and the recruitment of parkin (67, 68). The individual contributions of phosphorylations at S228 and S402 for PINK1 functions are not entirely clear. Functional PINK1 autophosphorylation of S228 in mammals is conserved in insects, where the homologous residue S346 in *Drosophila melanogaster* is needed for PINK1 activity and parkin translocation to mitochondria (69). The corresponding residue S205 in *Tribolium castaneum* is likewise an autophosphorylation site *in vitro* and affects substrate binding (70).

Once PINK1 is activated, it phosphorylates ubiquitin as well as the parkin Ubl at S65 (55, 56, 57). Even though S65 is conserved among various ubiquitin-like modifiers and domains as well as ubiquitin itself (55), the affinity of PINK1 for the parkin Ubl is 10-fold higher in comparison to monomeric ubiquitin (70). Charging the really interesting new gene (RING)-type E3 enzyme parkin with ubiquitin requires the sequential action of the E1 ubiquitin-activating enzyme and a cognate E2 conjugating enzyme. In the resting state, parkin is folded in an auto-inhibited manner (71). Parkin activation occurs upon binding to PINK1-phosphorylated [pS65]ubiquitin, which together with S65 phosphorylation in the parkin Ubl domain causes a conformational change to the active state (59, 72, 73). When such structural reorganization of the Ubl domain and E2 binding site of parkin has taken place, the E3 ligase parkin promotes the attachment of ubiquitin to specific MOM substrates during mitophagy (Fig. 2*B*). These events are antagonized by PTEN-L, the first discovered ubiquitin phosphatase (74).

### Priming steps for parkin translocation

At the onset of mitophagy, parkin is very efficiently translocated from the cytosol to the MOM of depolarized mitochondria, such that in model cells lines practically the entire immunofluorescence signal is found associated with mitochondria within an hour. Active PINK1 on the MOM is necessary and sufficient for this step (41). Abolishing PINK1 auto-phosphorylation at S228/S402 prevents parkin translocation after treatment of HeLa cells with the uncoupling agent carbonyl cyanide *m*-chlorophenyl hydrazone (CCCP) (67). Importantly, PD-linked PINK1 mutations also compromise this step and subsequent mitophagy (75, 76).

Pathogenic parkin mutations also impair mitophagy, either by preventing mitochondrial translocation to depolarized mitochondria or by failing to ubiquitylate its substrates (41, 52, 77). More specifically, targeting parkin catalytic activity by site-directed mutagenesis (C431S, C431F, and G430D) or rendering the PINK1 site unphosphorylatable (S65A) delay or compromise parkin translocation to the MOM after mitochondria depolarization (52, 57).

A straightforward model would posit that PINK1 accumulated on depolarized mitochondria would phosphorylate ubiquitin and parkin at the respective S65 residues, thereby activating the parkin ubiquitin ligase holoenzyme and enriching it on mitochondria by engagement with MOM substrates. Indeed, phosphorylation of parkin at S65 in the Ubl occurs without parkin reaching the MOM in cells expressing catalytically inactive parkin (C431S) (77). Thus, PINK1-mediated phosphorylation of the parkin Ubl S65 may trigger the induction of a fully active configuration of the parkin enzyme with [pS65]ubiquitin (78). Activation of parkin promotes its translocation from the cytosol to depolarized mitochondria. Accumulating on depolarized mitochondria, phospho-ubiquitin stimulated parkin strongly promotes the ubiquitylation of MOM proteins. Such ubiquitin molecules become substrates for further PINK1 phosphorylation, generating a feed-forward amplification loop at the MOM.

A specific subset of E2 ubiquitin-conjugating enzymes was identified that regulated parkin-dependent mitophagy (79, 80). Curiously, even the combined knockdown of these parkin coenzymes UBE2N, UBE2L3, and UBE2D2/3 only delays parkin recruitment and subsequent mitochondrial ubiquitylations and p62 localization to depolarized mitochondria, indicating additional regulatory steps for parkin recruitment and the process of mitophagy.

It seems hard to envision that cells rely for the intricate control of mitophagy on random vicinity of cytosolic ubiquitin and parkin with PINK1 accumulated on depolarized mitochondria. New insights into ubiquitin chain structures during mitophagy recently reveal similar relative amounts of mono-ubiquitylated and single-branched ubiquitin species in HeLa cells expressing catalytically active or inactive mutant (C431S) parkin, suggesting the presence of a mono-ubiquitin and single-branched ubiquitin coat in depolarized mitochondria (81). Likewise, recent global ubiquitylome analyses show similar amounts of mono-ubiquitylated and single-branched ubiquitylation species in induced neurons expressing both wild-type or nonphosphorylable parkin (S65 A) after 6 h of mitochondria depolarization (82). The mitochondrial E3 ligase MITOL mediates constitutive MOM protein ubiquitylation (83) that would become directly phosphorylated by PINK1 on the MOM of depolarized mitochondria, generating a phosphorylated MOM-ubiquitin coat available for parkin to ubiquitylate (Fig. 2*A*). In addition, the mitochondrial ubiquitin ligase MUL1 acts upstream of the PINK1/parkin pathway, restraining mitofusin-2 (MFN2) and hence maintaining mitochondrial morphology and ER-mitochondria contacts in stressed mature neurons (84).

### Parkin-mediated ubiquitylations

Parkin can ubiquitylate at least 2000 substrate proteins (85) in an apparently peptide sequence nonspecific manner (83). While the determination of parkin substrate specificity is quite unclear, there is a cadence of consistent and functionally important ubiquitylations during the process of mitophagy (Fig. 2, *B* and *C*), some of which are highlighted in the following.

Mitochondrial Rho GTPase (MIRO) proteins are important factors for mitochondrial motility. In parkin-expressing HeLa and SH-SY5Y cells, they are rapidly poly-ubiquitylated. Interestingly, MIRO-2 is not completely eliminated for at least 90 min, and although the unmodified MIRO-1 band vanishes with a half-life time of ≈20 min (86, 87), poly-ubiquitylated MIRO-1 persists for several hours. Thus, parkin-mediated ubiquitylations do not directly target MIRO proteins to proteasomal degradation (88). Rather, MIRO-1 could initially stabilize parkin on the MOM of damaged mitochondria and thus contribute to parkin recruitment (89). PINK1 phosphorylation of parkin Ubl (S65) is necessary for the activity of parkin towards MIRO-1 (86). A priming role for PINK1-mediated phosphorylation of MIRO-1 S156 (88) could not be confirmed (86, 87). Possibly the regulation of MIRO-1 turnover is more complex and involves additional phosphorylations at T298/299 (90). As the MIRO proteins drive mitochondrial trafficking, their subsequent degradation renders mitochondria more stationary for efficient autophagic removal (91). Moreover, by interaction with VPS13D, MIRO proteins regulate mitochondrial contacts with ER (92), possibly influencing the engagement with nascent phagophores.

Parkin recruited to depolarized mitochondria ubiquitylates MFN proteins and thereby marks them for rapid proteasomal degradation (93, 94). As a consequence of quick MFN degradation, the damaged mitochondrial network fragments, which is important for efficient mitophagy (95). Moreover, the PINK1/parkin-induced burst of MFN2 phospho-ubiquitylation triggers p97-mediated MFN2 complex disassembly and thus reduces the tethering of mitochondria with ER, facilitating mitophagy (96). On the other hand, parkin has also been reported to have an impact on mitochondrial fission by actively ubiquitylating the dynamin-related protein 1 (DRP1) and targeting it for rapid proteasomal degradation (97). In the context of mitophagy, where the mitochondrial network needs to be broken down (95), parkin-dependent degradation of the fission factor DRP1 would be counter-intuitive. Rather, PINK1 and parkin facilitate the recruitment of DRP1 and hence mitochondrial fission (98, 99). Indeed, proteomic studies identify strong parkin-dependent ubiquitylation sites of MFN1/2 after short times of mitochondrial depolarization, but hardly any consistent ubiquitylation events and no degradation of DRP1 seem to appear (82, 100). Thus, parkin-mediated poly-ubiquitylations directly downregulate fusion factors and more indirectly attract fission factors, which promote the mitochondrial fragmentation necessary for efficient autophagic removal.

Porins comprise a class of parkin substrates that was recognized early on in experiments knocking down VDAC1 (52). In mammals, these most abundant MOM proteins exist in three isoforms: voltage-dependent anion selective channel VDAC1-3. While the relative contribution of each individual VDAC for distinct steps of mitophagy was somewhat controversial (52, 101, 102), the cytosolic loops of all three VDACs were confirmed to contain lysine residues ubiquitylated by parkin (103). Ubiquitylation of VDAC1 as a paradigmatic parkin substrate was assessed after the gating step of MFN2 degradation and disruption of ER-mitochondria tethering upon induction of mitophagy (96). Furthermore, VDAC interacts with the mitochondria-associated isoform of hexokinase (HK2), which is implicated with parkin-mediated mitophagy as well (104, 105). Individual VDAC protein expression levels and ubiquitylation stoichiometry might differ with cell type and culture conditions, but VDACs are certainly targeted in parkin-mediated mitophagy, and it is possible that specific VDAC isoform PTMs affect selective aspects of mitochondrial quality control. For example, it was recently found that VDAC1 poly-ubiquitylations promoted mitophagy, whereas mono-ubiquitylation of VDAC1 at K274 rather conferred apoptosis (106).

In addition, parkin ubiquitylates and thus labels for degradation a number of additional MOM proteins, including subunits of the translocase of the outer membrane TOM20, TOM40, and TOM70 (107, 108). In fact, the TOM complex plays an important role in PINK1/parkin-mediated mitophagy (109), and the translocon is particularly targeted by one of the major deubiquitylating enzyme (DUB) modulators of parkin, the ubiquitin-specific protease USP30 (82, 110).

### Mitophagy ubiquitin linkage code

Ubiquitin contains seven lysine residues (K6, K11, K27, K29, K33, K48, and K63). Depending on the lysine used as a linkage for attaching the next ubiquitin molecule, several distinct ubiquitin chains may form that will differ on their acquired structure. While some chains have clear physiological consequences such as K48 or K63-linkage chains, which trigger proteasomal degradation or autophagy activation, respectively, less is known about the other poly-ubiquitin chains (111).

Even though the detailed linkage types and structures of the ubiquitin chains built on parkin substrates are unknown to date, initial studies focused on ubiquitin chain types during mitophagy showed that parkin-dependent mitophagy triggers the elimination of mitochondrial substrates by building K48-linked, proteasome-targeting poly-ubiquitin chains (107) as well as the formation of K63-linked poly-ubiquitin chains targeting for autophagy (80, 101). Moreover, K27 linkages were detected (52, 86). Indeed, parkin is able to build other types of ubiquitin-linked chains (K6, K11, and K27) apart from K48 and K63, not only linear but also branched poly-ubiquitin chains (77, 82, 112). The timing of parkin target selection and the linkage specificity of ubiquitin chain elongation are likely regulated by PTMs and regulatory factors that need to be further elucidated. How parkin writes the ubiquitin code for the mitophagy process is a key question for future studies.

### Role of DUBs

The MOM protein ubiquitylations formed during mitophagy are naturally subject to modulation by DUBs. The MOM residing DUB USP30 directly removes ubiquitin molecules attached by parkin on depolarized mitochondria, thus impeding parkin-dependent mitophagy progression (113, 114). Consequently, USP30 knockdown in a *Drosophila* model rescues defective mitophagy and fly behavior (113). Remarkably, USP30 acts poorly on K6-linked ubiquitins that are phosphorylated at the PINK1 site S65 (115, 116). Similarly, USP15 also has a direct impact on removing parkin-mediated mitochondrial ubiquitylations (117). Knockdown of USP15 in PD patient fibroblasts—which have reduced parkin levels—rescues mitophagy defects (117). Moreover, parkin itself is deubiquitylated on the MOM by USP33, notably at an apparently regulatory site (K435), leading to altered ubiquitin linkage activities and thus interfering with parkin recruitment and mitophagy (118). Conversely, USP8 actually promotes parkin-dependent mitophagy by preferentially removing autocatalytically formed K6-linked ubiquitin conjugates from parkin. In the absence of USP8, persistent K6-linked poly-ubiqutin chains on parkin interfere with the translocation of parkin to depolarized mitochondria and the promotion of mitophagy, possibly by affecting the functional interactions with PINK1-phosphorylated ubiquitin and/or interactions with ubiquitin-binding autophagy receptors (119).

Additional DUBs regulate mitophagy in a more indirect manner. USP35, which associates with mitochondria under normal conditions, translocates to the cytosol when mitochondria are depolarized (114). Perhaps the dissociation of the DUB USP35 from depolarized mitochondria is sufficient to facilitate actions of the ubiquitin ligase parkin. It remains unknown whether USP35 additionally regulates components of the autophagy machinery as was shown, *e.g.*, for USP33 (120). In fact, USP36 is a powerful regulator of mitophagy although it remains nuclear and does not translocate to mitochondria even under conditions of mitophagy (121). Instead, USP36 regulates the expression of ATG14L/beclin-1, possibly *via* epigenetic mechanisms.

Taken together, the process of parkin-mediated mitophagy is modulated at multiple steps: the direct parkin DUBs affect parkin activity and recruitment to damaged mitochondria in a facilitating (USP8) or repressive manner (USP33), the parkin-mediated MOM protein ubiquitylations can be subsequently removed (USP15, USP30) or left untouched (USP35), and specific effectors of the autophagy/mitophagy machinery can be indirectly regulated (USP36).

## Parkin-independent mitophagy

**Figure 3:**
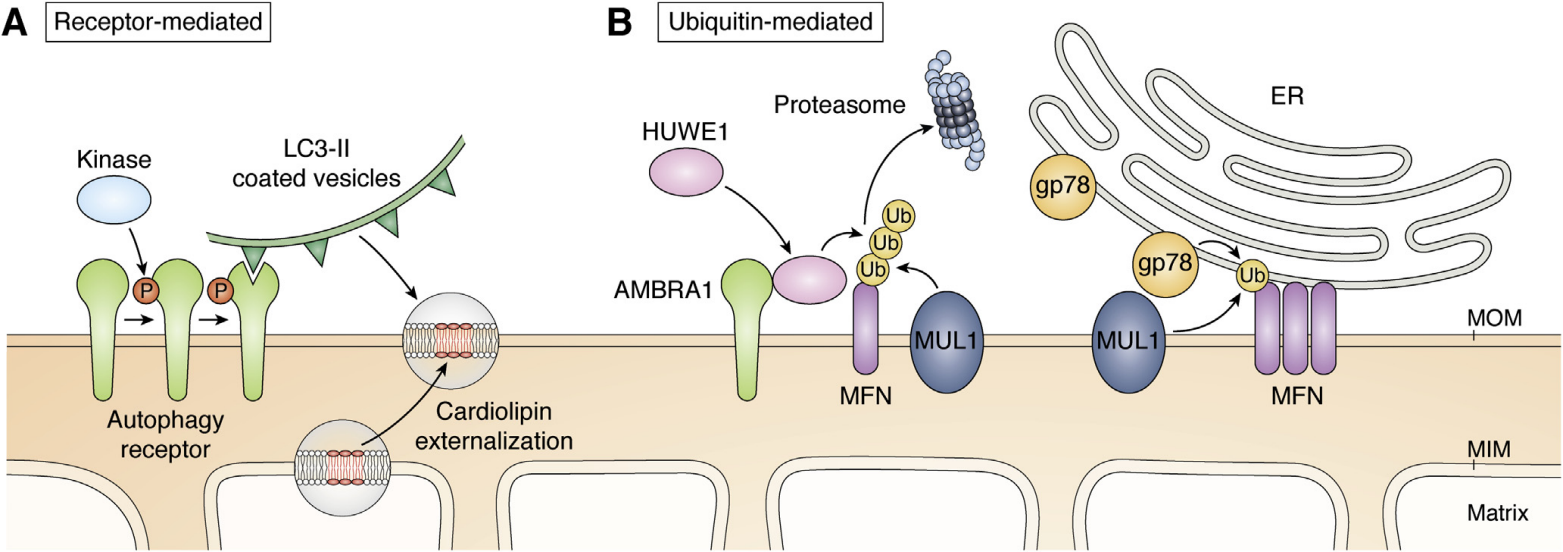
**Parkin-independent driven mitochondria elimination.***A*, receptor-mediated mitophagy depends on phosphorylation of MOM-anchored autophagy receptors (*i.e.*, NIX, BNIP3, AMBRA1, FUNDC1). Additionally, phospholipids such as cardiolipin can also act as autophagy receptors once they are externalized to the MOM under mitochondrial stress conditions. *B*, ubiquitin-mediated mitophagy involves parkin-independent E3 ligase ubiquitylations that specifically decorate mitochondria for subsequent autophagic removal. Such ubiquitylations may be performed by MOM-residing E3 ligases (*i.e.*, MUL1), cytosolic E3 ligasses that translocate to mitochondria upon mitochondrial stress conditions (*i.e.*, HUWE1) or ER-residing E3 ligases (*i.e.*, gp78) that, together with MOM-residing ubiquitin ligases, act on ER–mitochondrial contact sites, enhancing mitochondria fragmentation and facilitating specific autophagic elimination of mitochondria.

In addition to the PINK1/parkin-dependent mitophagy pathway described above, alternative parkin-independent pathways can recruit LC3 to mitochondria and promote their selective autophagic elimination (122, 123). Two different parkin-independent mechanisms can be distinguished: (1) receptor-mediated or (2) ubiquitin-mediated mitophagy (Fig. 3). In response to low oxygen (hypoxia) or mitochondrial uncoupling, receptor-mediated mitophagy will take place. Autophagy receptors residing at the MOM, which are regulated by phosphorylation or dephosphorylation, directly interact with LC3 molecules attached to the autophagosome, thereby promoting mitochondrial elimination through autophagy (124). BNIP3 and NIX are two MOM residing autophagy receptors that can be phosphorylated in their LIR domains (S17 or S34 and S45, respectively), promoting their interaction with LC3 (125, 126). Up to the present day, the kinases responsible for BNIP3 and NIX activation remain unknown. FUNDC1 is another MOM autophagy receptor that is auto-inhibited in basal conditions. FUNDC1 is intrinsically phosphorylated at Y18 and S13 by Src kinase and casein kinase 2, respectively. It becomes active under conditions of severe hypoxia, where the phosphoglycerate mutase family member 5—a mitochondrial serine/threonine-protein phosphatase dephosphorylates S13, allowing the interaction of the LIR domain with LC3 (127, 128). Additionally, ULK1 phosphorylation at S17 enhances LC3 interaction (25).

The activating molecule in beclin-1 regulated autophagy protein 1 (AMBRA1) was identified as a parkin-interacting protein mediating the propagation of mitophagy (129). Targeting of the autophagy receptor AMBRA1 to mitochondria directly couples to LC3 *via* a LIR motif and thus, launches mitophagy circumventing parkin and p62 (130). Like most autophagy receptors, AMBRA1 can be phosphorylated in the vicinity of the LIR motif (at S1014), which stimulates coupling to LC3B and promotes mitophagy. The inhibitor of nuclear factor κB (NF-κB) kinase-α was validated to phosphorylate its consensus sequence around S1014 -near the AMBRA1 LIR motif- upon mitophagy (131). How AMBRA1 and its associated factors accumulate on mitochondria for cargo-selective autophagic clearance remains to be further studied, as well as the potential regulatory role of Bcl-2 proteins interacting with AMBRA1 (132).

In addition to protein phosphorylations, other PTMs can also regulate autophagy. Protein lysine acetylation (133) is a straightforward part of the nutrient-sensing program, as all nutrients can be metabolized to acetyl-CoA under permissive cellular conditions. Specifically, general control of amino acid synthesis protein 5-like 1 (GCN5L1) is a positive regulator of protein acetylation in mitochondria (134). GCN5L1 deficiency leads to the association of LC3 and p62 with mitochondria and subsequent mitochondrial cargo-selective autophagy in an ATG-dependent but parkin-independent manner (135). In this experimental system using HepG2 cells, knockdown of the mitochondrial deacetylase sirtuin-3 (SIRT3) attenuated the mitochondrial recruitment of p62 and LC3-II promoted by GCN5L1 knockdown (135). In oxidatively stressed endothelial cells, induction of SIRT3 promotes mitophagy *via* deacetylation of the forkhead box protein O3, promoting the stimulation of gene batteries regulating mitochondrial biogenesis, fission/fusion, and the mitophagy effectors BNIP3, NIX, and LC3 (136). In oxidatively stressed endothelial cells, SIRT3 may directly impact on PINK1 and parkin (137). Moreover, SIRT3 can deacetylate optic atrophy protein 1 (OPA1), a mitochondrial fusion factor, which promotes mitochondrial integrity in stressed heart cells (138). Thus, the protein deacetylase SIRT3 could be a master regulator of mitochondrial turnover, quality control and mitophagy (139), both under normal conditions and in stressed cells with activated PINK1/parkin. However, the effects of SIRT3 are highly complex, and the molecular details for cargo-selective mitophagy in specific cell types remain to be resolved.

Mitochondria are rich in iron bound cytochromes and iron-sulfur clusters. Remarkably, the iron chelator deferiprone was a powerful inducer of PINK1/parkin-independent selective mitophagy even in PD patient fibroblasts (140). Deferiprone treatment causes the autophagy receptor FKBP8 on mitochondria to interact with LC3, thus initiating parkin-independent mitophagy (42, 141). Likewise, deferiprone induces mitochondrial clearance in myotubes by hypoxia-inducible factor 1-α mediated transcriptional induction of the LC3-binding autophagy receptors BNIP3 and NIX but, interestingly, instead of autophagic degradation in this case, mitochondria were secreted as extracellular vesicles (142). Taken together, iron homeostasis is important for PINK1/parkin-independent mitochondrial clearance.

Cardiolipin is a phospholipid normally residing at the MIM, which can be externalized under mitochondrial stress conditions by phospholipid scramblase-3. Such relocalization to the MOM allows cardiolipin to directly bind to LC3 (43), thus acting as a lipid kind of autophagy receptor in the regulation of parkin-independent mitophagy (122) (Fig. 3*A*).

Apart from parkin, other E3 ubiquitin ligases are also capable of decorating the MOM with the purpose of targeting them for specific autophagy elimination. The ubiquitin ligase homologous to the E6-AP carboxyl terminus, ubiquitin-associated and WWE domain-containing protein 1 (HUWE1) acts as an alternative to parkin, binding AMBRA1 upon mitophagy. Similar to parkin, HUWE1 can be recruited to depolarized mitochondria where it ubiquitylates MFN2 for proteasomal degradation (131). The ubiquitylation of MOM proteins may drive AMBRA1 activation and further mitophagy progression (131) (Fig. 3*B*).

Glycoprotein 78 (gp78), a ubiquitin ligase important for ER-associated degradation, has been shown to promote mitophagy in the absence of the ubiquitin ligase parkin (143). Like parkin, gp78 ubiquitylates MFN proteins to target them for proteasomal degradation, thereby promoting mitochondrial fragmentation. Interestingly, gp78 engages LC3 to ER-associated mitochondrial sites and promotes mitophagy in an ATG5-dependent manner. Gp78 levels are regulated by self-ubiquitylation as well as ubiquitylation by the ubiquitin ligase mahogunin RING finger protein 1 (MGRN1). Expression of a disease-causing prion protein disrupts the Ca^2+^-dependent interaction of MGRN1 with gp78 (144), preventing the degradation of gp78 and thus initiating mitophagy. It is possible that calcium stress affects turnover of ER–mitochondria contact sites, involving the alternative ubiquitin ligase gp78, also in a PINK1-dependent manner (145).

The mitochondrial ubiquitin ligase MUL1 shares many substrates with parkin and is able to compensate for PINK1/parkin loss of function in the context of PD, in *Drosophila* and mouse neurons, ubiquitylating MFN2 and promoting its degradation through the proteasome system (146). MUL1 can act together with MFN2 to control mitochondria morphology and ER–mitochondria contracts, and thus, it is suggested that when MUL1-MFN2 pathway is disrupted, the PINK1/parkin mitophagy pathway will be activated (84).

In addition to completely PINK1/parkin-independent mitophagy pathways, the ariadne-1 homolog in humans (ARIH1) promotes mitophagy in cancer cells independently of parkin but in a PINK1-dependent manner (147). ARIH1 is structurally very similar to parkin (148), but the expression pattern of these two ubiquitin ligases is quite different. Whereas parkin is widely expressed in neuronal cells and absent in many cancer cell lines (149), ARIH1 is highly expressed in various cancer cells (147). Interestingly, ARIH1 expression in cancer cells allows for apoptosis avoidance against chemotoxicity, suggesting that ARIH1-mediated mitophagy contributes to a chemotherapy resistance mechanism (147).

PINK1 and parkin are not only expressed in the nervous tissue but also in the muscle (150, 151). Both neurons and myocytes strongly depend on mitochondria. Thus, the complete autophagic elimination of mitochondria *via* the PINK1/parkin pathway commonly studied in cultured cell lines that satisfy their energy demands largely by glycolysis may not be reflected on all accounts in the brain, muscle, and heart *in vivo*. While DRP1-mediated mitochondrial fission is important for mitophagy in neurons and cardiomyocytes, parkin acts merely as a facilitating factor but not as essential regulator of mitophagy in neurons and cardiomyocytes (152). In this case, labeling of mitochondria with ubiquitin and the recruitment of p62 can occur even in the absence of the ubiquitin ligase parkin.

## Roles of accessory factors

### Mitochondrial fission/fusion factors

**Figure 4:**
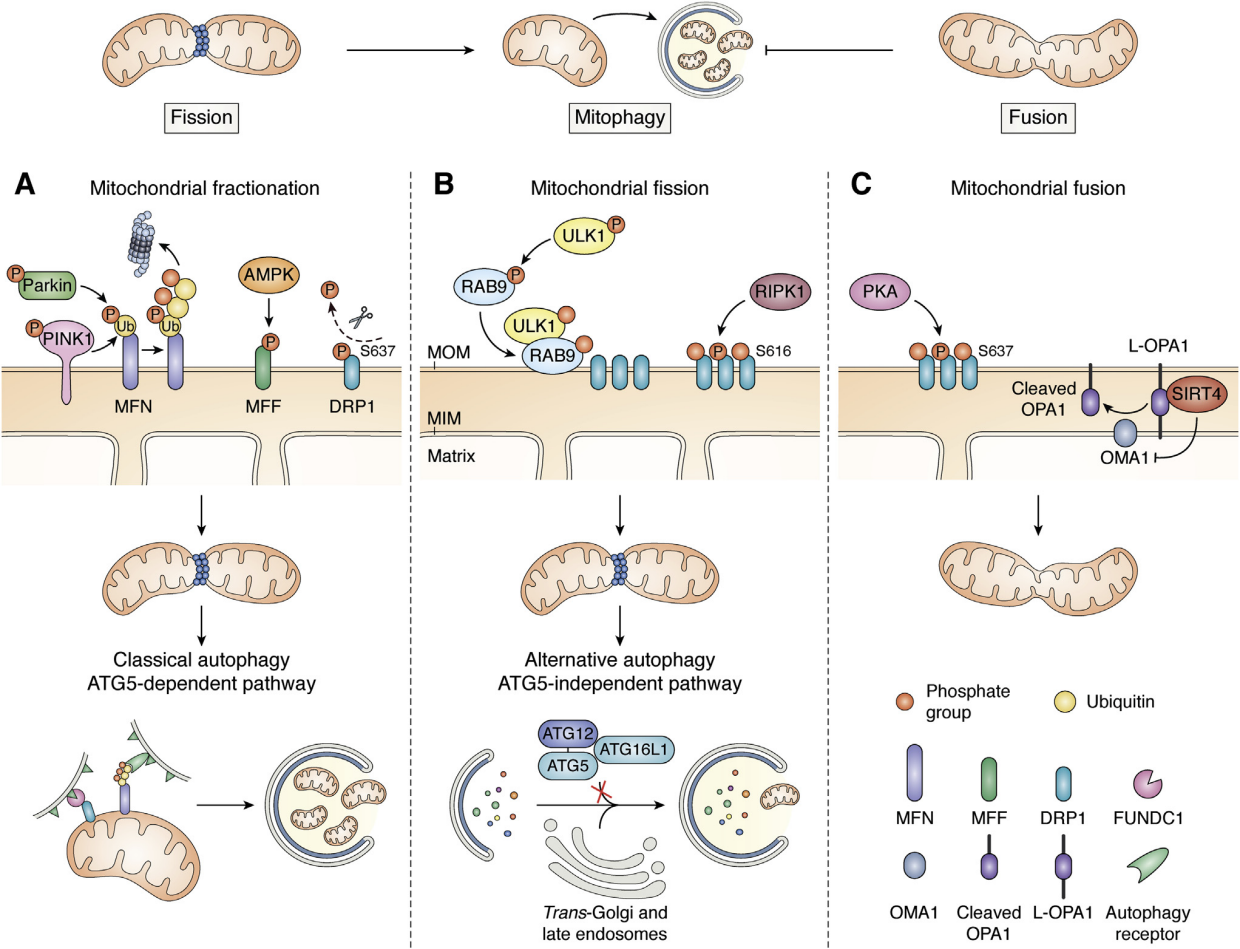
**Fission and fusion factors driving mitophagy.** Mitochondrial fragmentation triggers mitophagy while mitochondrial fusion has the opposite effect (*upper scheme*). *A*, mitochondrial fragmentation can occur through (1) parkin-dependent ubiquitylation of MFN, targeting MFN for proteasomal degradation, (2) phosphorylation of MFF by AMPK or (3) dephosphorylation of DRP1. Once mitochondria are fragmented, mitophagy is induced in an ATG5-dependent manner through recognition of ubiquitin chains *via* autophagy receptors or direct recognition of dephosphorylated DRP1 by FUNDC1. *B*, mitochondrial fission can be triggered by the RAB9/ULK1 complex, which indirectly influences the phosphorylation of DRP1. In this pathway, selective autophagy is activated in an ATG5-independent manner; the maturation of the phagosome is accomplished by the interaction of *trans*-golgi and late endosome membrane fusion. *C*, mitochondrial fusion can be driven through a change in DRP1 phosphorylation status by protein kinase A or by direct inhibition of L-OPA1 cleavage by SIRT4.

Mitochondrial fission was recognized early on to be important for mitophagy (95), likely to pinch off damaged fragments from the mitochondrial network for piecemeal mitophagy. Mitochondrial fission and autophagic removal are coupled by parkin *via* pathways associated with dephosphorylation of DRP1 at S637 in its GTPase domain (98). The mitochondrial dynamics protein of 51 kDa is an important cofactor for parkin/DRP1-mediated mitochondrial fragmentation and autophagic removal, with mitochondrial dynamics protein of 49 kDa and the mitochondrial fission factor (MFF) also contributing (98). The ULK1/*Atg1* kinase AMPK (14) also phosphorylates MFF at S172 (and S155) between the DRP1-interacting domain and the C-terminal mitochondrial targeting transmembrane domain (153) and may thus promote fission and mitophagy in cells suffering from energy crisis (Fig. 4*A*). Moreover, MFF expression is regulated by the RNA-binding translation repressor pumilio2 in an age-dependent manner (154). MFF/DRP1-fissioned mitochondria may then be delivered to the autophagolysosomal pathway by the ubiquitin-binding protein VPS13D (155). Moreover, MUL1 acts not only as a ubiquitin ligase but can also transfer the small ubiquitin-related modifier (SUMO) to DRP1. The mixed activities of MUL1 as a stabilizing DRP1-SUMO ligase and a destabilizing MFN2-ubiquitin ligase promote mitochondrial fission (156). Taken together, mitochondrial fragmentation and mitophagy are coordinated events ensuring optimal quality control for the mitochondrial network (Fig. 4).

Depletion of the mitochondrial fusion factors MFN and OPA1 causes hypofusion of the MOM and MIM, respectively (157). Downregulation of mitochondrial fusion factors would shift the mitochondrial morphology dynamics toward fragmentation. A major factor driving fragmentation of damaged mitochondria is the parkin-mediated ubiquitylation of MFN, targeting for proteasomal degradation of a key mitochondrial fusion factor (see above). Interestingly, depletion of MFN1 may also contribute to mitochondrial quality control in the female germline (158). Due to the relatively poor proofreading capacity of the mitochondrial DNA polymerase, mtDNA mutations accumulate that must be carefully removed from the maternal germline by purifying selection (159). As recently shown in *Drosophila*, mitochondrial fragmentation in early developing oocytes is necessary and sufficient for germline mtDNA selection (158). This process does not occur *via* the PINK1/parkin pathway. Rather the fragmented mutant mitochondria produced less ATP, which marked them for autophagic clearance involving *Atg1* and *BNIP3*, but not *Atg8a* (158). BNIP3-like targeting mitochondria to autophagy pathways independent of the ATG8 upstream activator ATG7 had been also suggested for the elimination of mitochondria from red blood cells (49, 160). An alternative macroautophagy pathway regulated by *Atg1*/ULK1 and beclin-1 was described in *Atg5*^−/−^/*Atg7*^−/−^ double-knockout mouse embryonic fibroblasts, where autophagosomes seem to be assembled in a RAB9-dependent manner by the fusion of isolation membranes with vesicles derived from the *trans*-Golgi and late endosomes (161) (Fig. 4*B*). Such a parkin- and ATG7-independent but ULK1- and RAB9-dependent alternative mitophagy pathway involving DRP1 protects cardiomyocytes against ischemic damage (162). ULK1 becomes phosphorylated prominently at the AMPK site S555 (14). In starved cardiomyocytes, (pS555)ULK1 phosphorylates RAB9 at S179, which leads to the formation of an (pS555)ULK1/(pS179)RAB9 complex on mitochondria, that recruits the receptor-interacting protein kinase 1, which in turn mediates the fission-promoting phosphorylation of DRP1 at S616 (162). This mitochondrial fission/mitophagy complex eventually associates also with the autophagolysosomal fusion SNARE syntaxin17. The regulation of mitochondrial cargo selectivity of alternative macroautophagy in developing and stressed cells remains to be studied in detail.

MFN1 is the major MOM fusion factor and OPA1 regulates MIM fusions (157). Similar to MFN depletion, OPA1 deficiency results in mitochondrial fragmentation due to hypofusion, which facilitates mitophagy in optic atrophy patient fibroblasts (163). Interestingly, the mitochondrial autophagy receptor FUNDC1 not only couples to LC3 during hypoxia-mediated mitophagy (164), but also promotes mitochondrial fragmentation by shifting the binding of FUNDC1 from OPA1 to the mitochondrial fission factor DRP1 (128). This shift is regulated by FUNDC1 dephosphorylation at S13 near the LIR motif. Thus, mitochondrial fission and autophagy engagement may be efficiently integrated cellular processes connected by multifunctional autophagy receptors, subject to regulation of PTMs.

Conversely, hyperfusion spares mitochondria from degradation upon starvation-induced autophagy (165, 166). In this case, phosphorylation of DRP1 prevents the mitophagy-facilitating fragmentation of mitochondria. In addition, mitochondrial fusion is achieved by preventing cleavage of the fusion-promoting long isoform L-OPA1 by the endopeptidase overlapping with the mitochondrial matrix ATPase associated with diverse cellular activities (m-AAA) protease 1 (OMA1) (Fig. 4*C*). Regulated expression of OMA1 may modulate mitophagy *via* OPA1-mediated morphology dynamics (167, 168). The mitochondrial sirtuin SIRT4 was reported to bind and elevate levels of L-OPA1, suppressing mitophagy by mitochondrial elongation (169). It remains to be further elucidated which protein deacetylations mediate sirtuin effects on mitochondrial metabolism and cargo-selective autophagy (see also above).

### Bcl-2 family members

**Figure 5:**
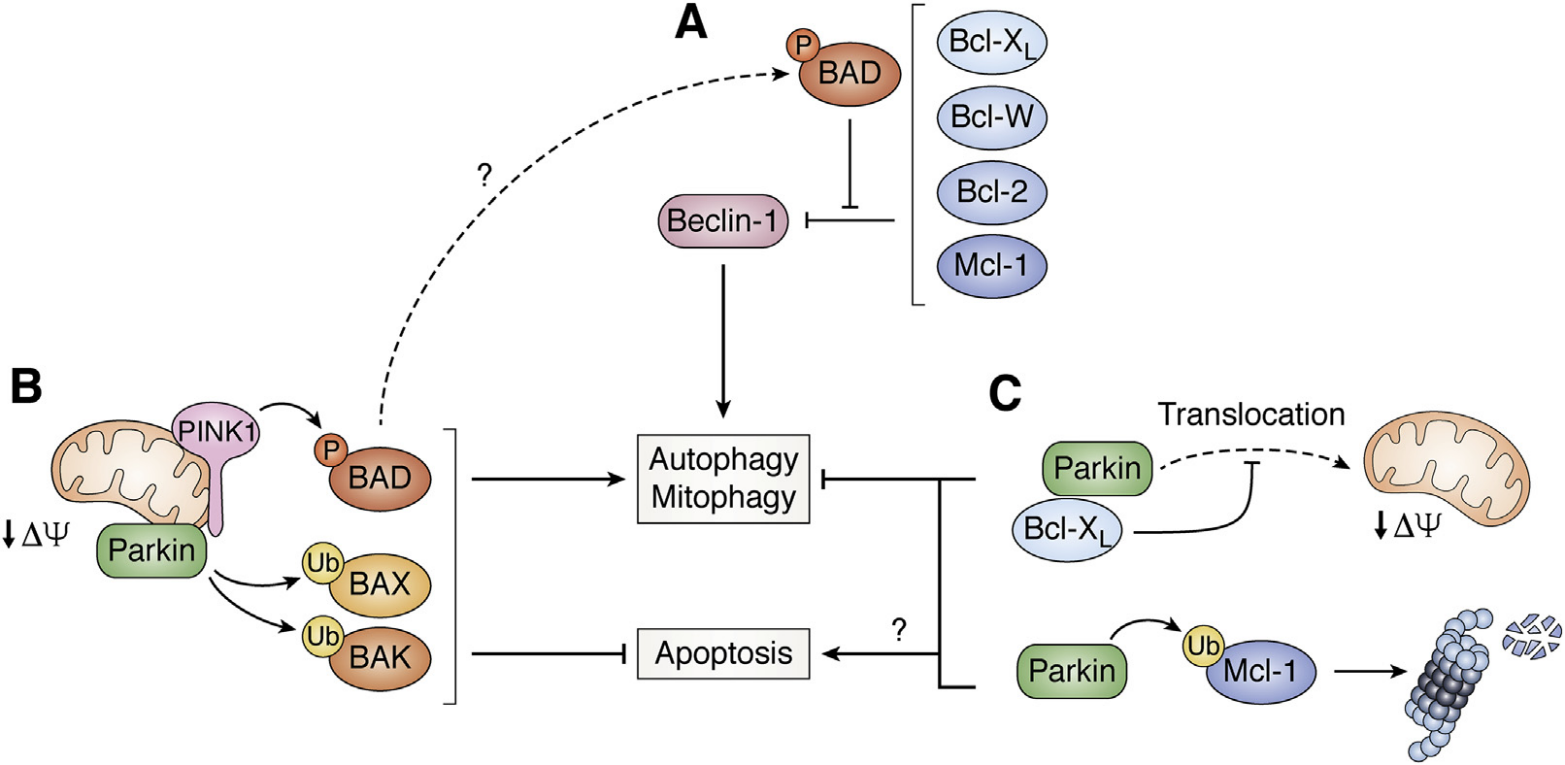
**Bcl-2 like proteins influence on the activation of autophagy and apoptosis.***A*, Bcl-2 like proteins can interact with beclin-1, inhibiting its autophagy function. BAD is able to relieve this repression, interfering with these interactions. *B*, under mitochondrial stress conditions, PINK1 phosphorylates BAD and parkin ubiquitinates BAX and BAK, inhibiting their proapoptotic functions and promoting mitophagy. It is possible—though not yet proven—that phosphorylation of BAD inhibits its proapoptotic functions also in this case. *C*, mitophagy can be inhibited by parkin-dependent ubiquitylation of the antiapoptotic factor Mcl-1 or direct interaction of Bcl-X_L_ with parkin, which directly inhibits parkin translocation to mitochondria. It remains to be elucidated if both parkin-dependent ubiquitinations eventually trigger cell apoptosis and through which specific pathway. *Arrowheads* indicate activation, *hammerheads* indicate inhibition, *dashed arrows* indicate translocation.

The MOM, which connects to the autophagy machinery during mitophagy, is strongly influenced by members of the Bcl-2 family. Bcl-2 proteins are key regulators of MOM permeability controlling the intrinsic, mitochondrial apoptosis pathway. It is becoming increasingly clear that Bcl-2 proteins have functions beyond their involvement in the regulation of apoptosis (170), including mitochondrial physiology and autophagy (Fig. 5). For example, interactions of anti-apoptotic Bcl-2 family members with beclin-1 repress autophagy (Fig. 5*A*). Specifically, beclin-1 contains a Bcl-2 homology 3 domain (BH3) and hence binds to antiapoptotic proteins such as Bcl-2, Bcl-W, Bcl-X_L_, and the induced myeloid leukemia cell differentiation protein Mcl-1 (171, 172). Conversely, the BH3-only Bcl-2 associated agonist of cell death (BAD) disrupts the interaction of antiapoptotic Bcl-2 proteins with beclin-1, thereby antagonizing autophagy repression (171).

Bcl-2 family members are also affected in PINK1/parkin-dependent mitophagy. In CCCP-treated neurons, the upregulated PINK1 phosphorylates ubiquitin S65 and with a similar time course multiple serine residues of the Bcl-2 associated agonist of cell death, suppressing its proapoptotic translocation to mitochondria (173). Parkin can ubiquitylate the proapoptotic Bcl-2 associated protein X (174, 175) and Bcl-2 homologous antagonist/killer (BAK), the resulting proteasomal degradation of these proapoptotic Bcl-2 family members would prevent apoptosis to allow cells to cope with the mitophagic stress (176) (Fig. 5*B*). On the other hand, the ubiquitin ligase parkin can also target the prosurvival Mcl-1 for proteasomal degradation (177). Thus, parkin may promote mitochondrial quality control by triggering mitophagy while suppressing apoptosis by ubiquitylation-mediated proteasomal targeting of proapoptotic Bcl-2 family members but when the damage cannot be relieved, parkin-mediated degradation of Mcl-1 may switch cell fate to apoptosis (178) (Fig. 5*C*). Moreover, the prosurvival protein Bcl-X_L_ actually represses mitophagy by direct binding to parkin and hindering its translocation to mitochondria in various stressed cells (179, 180, 181). In addition, PINK1 can phosphorylate Bcl-X_L_ on the MOM of CCCP-treated SH-SY5Y cells, not regulating mitophagy but rather suppressing apoptosis by preventing the proapoptotic cleavage of Bcl-X_L_ (182). Taken together, the cytoprotective enzymes PINK1 and parkin are distinctively interconnected with anti- and proapoptotic Bcl-2 family members and mitochondria at the crossroads of mitophagy and apoptosis (183), but the overall mechanism remains to be elucidated.

## Large-scale analyses of mitophagy-related processes

Integrative (multiomics) studies address biological processes in which mitochondria play crucial roles, such as mitophagy and apoptosis, with a strong disease-related context (82, 103, 184). Development and improvement of biochemical and bioinformatic tools allow the analysis of PTMs of mitochondrial proteins, such as protein phosphorylations or ubiquitylations (185, 186, 187). In the analyses of disease-related changes in the ubiquitylome, further development of existing methods enables the investigation of the ubiquitin chain architecture. For example, the “Ub-clipping” technique revealed that parkin mainly generates ubiquitylations that are mono-ubiquitylated or only consist of a short chain with a preference for distal ubiquitin phosphorylation (81).

The integration of several layers of -omics studies is essential to gain a more complete view of mitochondrial biology. Recently such integrative studies were done in the field of systems biology, *e.g.*, for modeling biological processes involving mitochondria. For example, combination of transcriptomics, proteomics, and metabolomics data led to the identification of the activating transcription factor 4 as a key regulator of mitochondrial stress response in different mammalian cell lines (188). Integration of transcriptomics and proteomics data led to the identification of adaptive changes in the protein expression after blocking the TOM complex import machinery in yeast (189). Combination of multiomics datasets with information on multiple cellular pathways allows even broader analysis of functional relationships. An example is the switch between mitophagy and cell death, which represents a complex and so far only poorly understood process (190). Another example of the importance of -omic studies as a fundament for subsequent analysis is the VDAC1 ubiquitylome. Ordureau *et al*. (103) described parkin-dependent ubiquitylation events of multiple lysine residues on VDAC1 in a quantitative ubiquitylome analysis. VDAC1 serves as a poly-ubiquitin anchor during mitophagy but can become an essential part of the mitochondrial permeability transition pore in apoptosis. The choice of apoptosis or mitophagy may be driven by parkin-dependent mono- or poly-ubiquitylation on VDAC1 K274 (106). As mentioned above, the decision between mitophagy and apoptosis is also regulated by functional interactions of PINK1 and parkin with antiapoptotic and proapoptotic Bcl-2 family members. Large-scale mass spectrometric determinations of the phosphoproteome (173) and ubiquitylome (100) help to appreciate the contribution of PTMs in the complex interplay between mitophagy and apoptosis. More advanced technologies will allow for even better integration of multiomics data to ultimately enhance the diagnosis and therapy of mitochondrial dysfunction in human diseases (191, 192).

## Mechanisms regulating mitochondrial cargo-selective autophagy

Autophagy receptors connecting to-be-degraded cargo to the autophagy machinery are logical regulatory effectors of selective autophagy. The tumor necrosis factor receptor–associated factor family member associated NF-κB activator-binding kinase 1 (TBK1) phosphorylates several autophagy receptor proteins on multiple, autophagy-relevant sites. When optineurin interacts with ubiquitin chains on MOM proteins during mitophagy, the constitutively optineurin-bound TBK1 is recruited to mitochondria and becomes activated (193, 194). TBK1-mediated phosphorylation of optineurin at S473 (and S513) in the ubiquitin-binding region further enhances the affinity of optineurin for poly-ubiquitin chains (193)—including those phosphorylated by PINK1 (194), thus promoting PINK1/parkin-dependent mitophagy in a feed-forward manner (Fig. 2*C*). Moreover, TBK1-catalyzed phosphorylation of S177—near the optineurin LIR motif—stabilizes the binding of optineurin to ubiquitylated mitochondria (39, 194), in addition to an influence on LC3 recruitment (193). Curiously, the LIR motif of optineurin (and another mitophagy adaptor, NDP52) was recently found to be dispensable for PINK1/parkin-mediated LC3/GABARAP recruitment and selective mitophagy (195). Rather, the LIR motif mediates secondary, ubiquitin-independent recruitment of optineurin and NDP52 to nascent autophagosomes, amplifying ULK1-regulated mitophagy. NDP52 tethering to peroxysomes and mitochondria, respectively, is sufficient to promote the recruitment of the ULK1/FIP200 complex facilitated by TBK1 (196). The function of NDP52 is the local recruitment of ULK1 for cargo-selective pexophagy, mitophagy, and xenophagy (196, 197).

## Conclusions and outlook

Selective removal of mitochondria—mitophagy—is essential for cellular quality control during stress and disease as well as in development. Thus, impaired mitophagy situations underlie the pathogenesis of a number of chronic diseases such as cancer, cardiovascular diseases, and neurodegenerative diseases, with a particular link to PD *via* the recessive gene products PINK1 and parkin. As outlined in this review, there are multiple pathways capable of promoting mitochondria elimination. While they all differ on their activation and action mode, all of them depend on accurate PTM regulations for their proper progression and functionality. Because it is essential to understand the molecular mechanisms that trigger mitophagy in all its possible ways, here we gathered the most important PTMs that influence mitochondria targeting mechanisms as well as autophagy-related processes, culminating with mitochondrial elimination.

Enormous research efforts in the past two decades identified the core elements of autophagy (Fig. 1), stress-related PINK1/parkin-dependent mitophagy (Fig. 2), receptor-mediated mitophagy during development (Fig. 3), and the involvement of fission/fusion factors (Fig. 4) and Bcl-2 family members (Fig. 5). Now is an exciting time to build on this knowledge to unravel the regulatory mechanisms that govern the different mitophagy pathways. Recent advances and future improvements in large-scale multiomic analyses will help in this endeavor.

The AMPK-ULK1 axis for starvation-induced autophagy is well established. However, much of the PINK1/parkin mitophagy knowledge is derived from cancer cell lines treated with bulk uncoupling agents. Due to the Warburg effect, though, cancer cells treated with uncoupling agents do not experience the same degree of energy crisis as cells subjected to nutrient starvation. The autophagy machinery is clearly activated during PINK1/parkin-mediated autophagy, but are the upstream mechanisms exactly the same as in nutrient starvation models? The recent discovery of a novel phosphorylation site in ULK1 regulating the alternative, ATG5-independent autophagy (198) is a precedent showing that even long-known players can exert novel modes for distinct autophagy pathways. Moreover, abrupt complete breakdown of ΔΨ_m_ is obviously an idealized experimental condition with questionable relevance particularly to postmitotic neurons (199). The recent development of fluorescent reporter systems confirms the occurrence of mitophagy in neurons, but further research with more physiological systems and *in vivo* aging models is needed to clarify which mitophagy regulatory elements are global and which are more specific in distinct stressed organs.

A key question is how the various ubiquitin ligases establish the ubiquitin code on to-be-degraded mitochondria, and how the autophagy machinery deciphers this code. Some substrates appear straightforward, such as the proteasome-targeting K48-linked poly-ubiquitylation of MFNs. The differential dynamics of TOM ubiquitylations suggest a more regulatory impact of the mitochondrial protein import machinery for the insertion of PINK1 into the MOM and the execution of mitophagy. The complex ubiquitin linkages that occur throughout the time course of mitophagy (103) might indicate a meaningful pattern of ubiquitin chains that have a signaling capacity much beyond simple protein targeting. Methodological advances to detect ubiquitin linkages and topologies (200) are instrumental in this regard. Another issue is the experimental modeling of ubiquitylated proteins. While protein serine/threonine phosphorylations can be mimicked faithfully in many cases by site-directed mutageneis to aspartate/glutamate and substitution with glutamine resembles acetyl-lysine to a certain degree, protein ubiquitin modifications cannot be simply mimicked by site-directed mutagenesis. Nevertheless, expanding the genetic code with amber suppression at a desired residue combined with sortase-mediated transpeptidation may provide a first step toward introducing specific ubiquitylated protein species into cells and study their functions (201).

Another issue to be fully resolved is the sequential dynamics of PTMs on the MOM and accessory factors. While the initiation phase is reasonably well understood (*e.g.*, PINK1 phosphorylation of ubiquitin S65 and the activation of parkin, proteasome-targeting MFN ubiquitylations), it is less clear if there are later checkpoints. Are there more convergence points of PINK1/parkin labeled mitochondria with components of the autophagy machinery (for example, the ULK1 complex, ER–mitochondria contacts, phagophores, etc.), and how would those comprise checkpoints regulating mitophagy throughout the entire time course? Are priming phosphorylations and/or dephosphorylations required for subsequent ubiquitin modifications and substrate selection of the E3 ligase(s)? Would these be exclusively mediated by the currently known ubiquitin kinase PINK1 and phosphatases, respectively? Is the cascade of ubiquitin ligases MUL1-MITOL-parkin established or would there be more E3 ligases involved, also in feedback with distinct autophagy regulators? Does this have an impact on the types of (phospho)ubiquitin chains built on MOM substrates and the writing of a putative mitophagy ubiquitin code? And finally, are the regulatory PTMs restricted to the interface of the MOM with autophagy membranes? The nuclear USP36 is a strong regulator of mitophagy controlling the expression of ATG14L (121). Could [pS65]ubiquitin act as a signal transducer from PINK1 accumulated on damaged mitochondria into the nucleus (202), affecting epigenetic and transcriptional regulators of stress-induced mitophagy?

Are protein phosphorylations, acetylations, and ubiquitin modifications the only PTMs regulating mitophagy? Recent evidence points to glycosylations. N-glycanase 1 (NGLY1) deficiency is a rare congenital disorder leading to global developmental delay and a multisystem syndrome including neuropathy. Interestingly, NGLY1 knockdown significantly impaired mitophagy in HeLa cells stably expressing parkin (203). While NGLY1 can regulate transcription of proteasomal genes *via* deglycosylation of the nuclear factor erythroid 2-like 1, a very recent study identified AMPKα as a novel NGLY1 target integrating energy sensing and mitochondrial homeostasis (204). It will be interesting to further study glycosylations in the regulation of autophagic turnover of mitochondria.

More questions arise when thinking of the cargo-selective specificity of (1) alternative macroautophagy in developing and stressed cells in general or (2) autophagy adaptors in particular, which may or not be driven by PTM modulations. More specifically, it still remains to be elucidated (1) which implications have sirtuin actions on mitochondrial metabolism and cargo-selective autophagy and (2) how do AMBRA1 and its associated factors accumulate on mitochondria for cargo-selective autophagy removal. Another interesting point to be addressed is how Bcl-2 like proteins may regulate autophagy adaptors (*i.e.*, AMBRA1) or other E3-ligases (*i.e.*, HUWE1, MITOL, MUL1). Increasingly sophisticated multiomic screens and future developments in biotechnological methodology combined with hypothesis-driven functional validations of PTM targets all along the mitophagy pathways will undoubtedly advance the comprehensive understanding of this vital process.

## Conflict of interest

The authors declare that they have no conflict of interest with the contents of this article.
